# Case Report: Three cases involving the use of endovascular intervention for artery of Percheron infarction with a unilateral fetal-type posterior cerebral artery

**DOI:** 10.3389/fsurg.2026.1798056

**Published:** 2026-07-01

**Authors:** Yue Xu, Minjia Yang

**Affiliations:** 1 Department of Neurology, The First Hospital of Putian City, Putian, China; 2 School of Clinical Medicine, Fujian Medical University, Fuzhou, China

**Keywords:** artery of Percheron, clinical features, endovascular intervention, fetal-type posterior cerebral artery, prognosis

## Abstract

**Background:**

Occlusion of the artery of Percheron (AOP) in the context of a unilateral fetal-type posterior cerebral artery (fPCA) is a rare cerebrovascular etiology for bilateral thalamic infarction and is associated with distinctive clinical features. However, most of the previous literature on Percheron artery infarction consists of case reports without involvement of the fPCA. Here, we present and discuss the clinical characteristics of three cases of AOP infarction with a unilateral fPCA.

**Case presentation:**

All three patients presented with acute and profound impairment of consciousness and pupillary dysfunction. Neuroimaging [cranial magnetic resonance imaging (MRI) and computed tomography (CT)] revealed bilateral paramedian thalamic infarctions. Cerebrovascular studies confirmed a unilateral fPCA. Basilar artery occlusion was identified in Cases 1 (77-year-old woman) and 2 (62-year-old woman), while Case 3 (68-year-old man) exhibited severe stenosis of the left posterior cerebral artery (PCA) P1 segment. All three patients had large vessel lesions with an onset within 6 h. All patients underwent endovascular treatment without perfusion examinations. Outcomes were poor in Cases 1 and 3, while Case 2 showed neurological improvement.

**Conclusion:**

AOP infarction, although rare, warrants suspicion in patients presenting with an acute impairment of consciousness, pupillary abnormalities, and vertical gaze palsy, particularly in the absence of prominent limb weakness. The presence of a unilateral fPCA provides a critical diagnostic clue when bilateral paramedian thalamic infarction is confirmed radiologically (with or without midbrain involvement). Early neuroimaging, particularly MRI, is paramount for timely diagnosis. Although endovascular therapy may benefit selected cases, the overall prognosis remains guarded.

## 1 Introduction

The artery of Percheron (AOP) is an uncommon anatomical variation that originates from the P1 segment of one posterior cerebral artery (PCA) and supplies blood to both paramedian thalami (1). AOP infarction accounts for approximately 0.1%–2% of all ischemic strokes and 4%–18% of thalamic strokes (2, 3). Because the AOP is typically too small for direct visualization on standard cerebrovascular imaging [magnetic resonance angiography (MRA), computed tomography angiography (CTA), or digital subtraction angiography (DSA)], diagnosis is primarily based on the characteristic pattern of infarction and the clinical presentation (4, 5). The presence of a unilateral fetal-type posterior cerebral artery (fPCA) provides stronger indirect evidence for the AOP variant when bilateral paramedian thalamic infarction is observed (6). However, few studies have examined Percheron artery infarction with a unilateral fPCA. In this report, prepared in accordance with the CARE guidelines, we present three cases of AOP infarction with a unilateral fPCA. Our aim was to enhance recognition and understanding of this condition.

## 2 Case reports

The key clinical features, imaging findings, treatment strategies, and outcomes of the three cases are summarized in Table 1.

**Table 1:** Summary of three patients with artery of Percheron infarction with a unilateral fetal-type posterior cerebral artery.

Case number	Age/gender	Occlusion site	DPT	DRT	Treatment	Outcome
Case 1	77/F	Distal basilar artery with right PCA occlusion	176 min	120 min	EVT + IVT	Death
Case 2	62/F	Basilar artery occlusion	75 min	95 min	EVT	Favorable outcome (mRS 2 at 90 days)
Case 3	68/M	Left PCA (P1) stenosis + left vertebral artery (V4) stenosis	Not available	Not applicable	Balloon angioplasty	Death

### 2.1 Case 1

A 77-year-old woman with a history of hypertension presented with a sudden onset of unconsciousness. On admission, examination revealed coma [Glasgow Coma Scale (GCS) score: 6]. The pupils were bilaterally miotic (1.0 mm), without a left pupillary light reflex and a sluggish response on the right. Bilateral Babinski signs were negative. The National Institutes of Health Stroke Scale (NIHSS) score was 40. Cranial magnetic resonance imaging (MRI) and MRA revealed acute infarcts in the bilateral thalami and cerebellum, ill-defined flow in the distal basilar artery, and non-visualization of the right PCA P1 segment. Intravenous alteplase thrombolysis was administered at a dose of 0.9 mg/kg (total dose 54 mg), followed by mechanical thrombectomy approximately 90 min later. Mechanical thrombectomy was performed using a stent retriever-based technique combined with aspiration. One pass was required. Continuous aspiration through an intermediate catheter retrieved a dark red thrombus (approximately 3 mm ×  2 mm). The door-to-puncture time was 176 min, and the puncture-to-recanalization time was 120 min. Angiography of the left vertebral artery confirmed partial occlusion of the distal basilar artery, occlusion of the right PCA and right superior cerebellar artery, and hypoplasia of the left PCA P1 segment. Angiography of the left common carotid artery confirmed a left fPCA (Figure 1). Postoperative management included statin therapy, intravenous fluids, and neuroprotective agents. Follow-up MRI on postoperative day 2 revealed hemorrhagic transformation within the cerebellar infarct and brainstem compression, requiring intensified osmotic therapy. The patient's level of consciousness showed no significant improvement. She was discharged on postoperative day 3 in response to a family request. The patient died several days after discharge.

**Figure 1:**
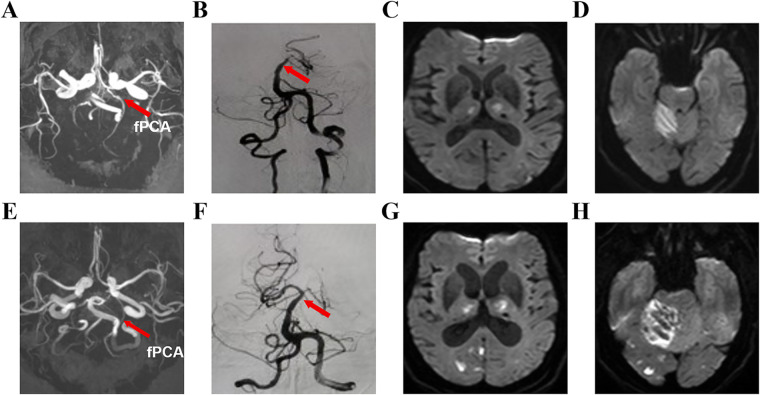
Pre-intervention and post-intervention. **(A)** Pre-intervention MRA, left fetal-type PCA, non-visualized right P1, stenotic P2; **(B)** pre-intervention DSA, subtotal basilar occlusion (TICI 1), occluded right PCA/SCA; **(C,D)** Pre-intervention DWI, acute bilateral thalamic and right cerebellar infarcts; **(E)** post-intervention MRA, patent right PCA; **(F)** post-intervention DSA, basilar recanalization (TICI 3); and **(G,H)** post-intervention MRI, cerebellar infarct expansion with hemorrhagic transformation causing brainstem compression. MRA, magnetic resonance angiography; PCA, posterior cerebral artery; DSA, digital subtraction angiography; TICI, thrombolysis in cerebral infarction; SCA, superior cerebellar artery; DWI, diffusion weighted imaging; MRI, magnetic resonance imaging.

### 2.2 Case 2

A 62-year-old woman with a history of hypertension, aortic valve replacement, permanent pacemaker implantation, and left hip fracture presented with a sudden loss of consciousness after using the toilet. She had been taking warfarin for long term; however, international normalized ratio (INR) monitoring had been inconsistent. Examination on admission revealed coma (GCS score: 6). The pupils were bilaterally equal and miotic (1.0 mm), with sluggish light reflexes. There was no movement of the left limbs in response to painful stimuli, and the left Babinski sign was positive. The NIHSS score was 31. Head and neck CTA revealed occlusion of the basilar artery, occlusion of the right internal carotid artery, and a left fPCA (Figure 2). Due to the unavailability of coagulation parameters, thrombolysis was withheld, and mechanical thrombectomy (basilar artery) was performed directly using an aspiration-assisted technique. Two passes were required to retrieve multiple thrombi of varying consistency. The door-to-puncture time was 75 min, and the puncture-to-recanalization time was 95 min. Postoperative therapy included osmotic agents, anticoagulation (adjusted according to INR), neurotrophic agents, intravenous fluids, antibiotics, and deep vein thrombosis prophylaxis. On postoperative day 3, the patient developed respiratory failure, requiring transient intubation until day 5. She was discharged in an improved condition on postoperative day 26. After 90 days, the prognosis was favorable, with a modified Rankin Scale (MRS) score of 2.

**Figure 2:**
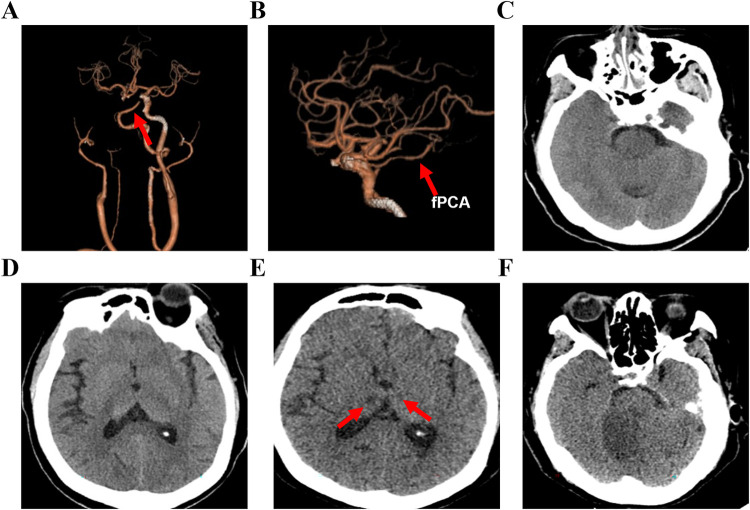
Pre-thrombectomy and post-intervention imaging. **(A,B)** Pre-thrombectomy imaging: cranial CTA showing occlusion of the left fetal-type posterior cerebral artery, distal basilar artery, and right internal carotid artery; **(C,D)** pre-thrombectomy imaging: non-contrast cranial CT showing no evidence of infarction; and **(E,F)** post-intervention imaging: non-contrast cranial CT showing infarction in the bilateral thalami and the right cerebellar hemisphere. CTA, computed tomography imaging; CT, computed tomography.

### 2.3 Case 3

A 68-year-old man with a history of hypertension and previous treatment for tuberculosis presented with a sudden-onset loss of consciousness. Neurological examination revealed moderate coma, bilaterally equal pupils (3.0 mm), absent light reflexes, and negative bilateral Babinski signs. The NIHSS score was 31, and the GCS score was 6. MRI/MRA of the head and neck revealed acute infarcts in the bilateral thalami and right occipital lobe, severe stenosis of the left PCA P1 segment, and a right fPCA. Cerebral angiography revealed a right fPCA, two severe focal stenoses in the left vertebral artery V4 segment, and severe stenosis of the left PCA P1 segment. Intravenous tirofiban infusion (0.25 mg/h) was initiated, given the presence of severe intracranial stenosis and a high thrombotic risk. Staged balloon angioplasty was subsequently performed to target the two stenotic lesions in the vertebral V4 segment (Figure 3). The decision not to intervene with the PCA P1 segment was deliberate. Given the small caliber and anatomical complexity of the P1 segment, as well as the increased risk of vessel injury and hemorrhagic complications associated with endovascular manipulation in this region, we considered that direct intervention was high risk. Therefore, treatment was focused on improving posterior circulation perfusion by performing angioplasty of the vertebral artery. Postoperative management included dual antiplatelet therapy (aspirin and clopidogrel), administration of a statin, and intravenous hydration. On postoperative day 5, the patient remained unconscious (an obtunded state), with absent ocular movements and pupils measuring 2.0 mm without light reflexes. The patient died 2 weeks after discharge from the hospital. It was not possible to perform follow-up neuroimaging.

**Figure 3:**
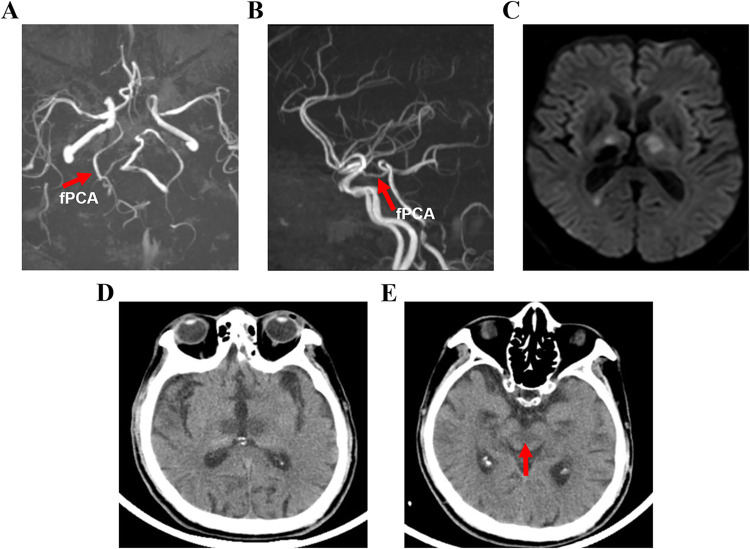
Pre-thrombectomy and post-intervention imaging. **(A–C)** Pre-thrombectomy imaging: brain MRI demonstrating acute infarction in the bilateral thalami and the right occipital lobe. Severe stenosis is present at the origin of the left PCA. The right PCA exhibits a fetal-type configuration. **(D,E)** Non-contrast brain CT scan showing infarction involving the bilateral thalami and midbrain. MRI, magnetic resonance imaging; PCA, posterior cerebral artery; CT, computed tomography.

## 3 Discussion

Direct visualization of the AOP is often not feasible; therefore, diagnosis is typically based on characteristic imaging patterns in conjunction with clinical features. AOP infarction classically manifests with acute impairment of consciousness, oculomotor disturbances (notably vertical gaze palsy), and pupillary abnormalities. Lazzaro et al. (2) established a classification system for AOP infarction patterns, categorizing them into four types: Type I (bilateral paramedian thalamic infarction with midbrain involvement), Type II (bilateral paramedian thalamic infarction without midbrain involvement), Type III (bilateral paramedian thalamic infarction with anterior thalamic and midbrain involvement), and Type IV (bilateral paramedian thalamic infarction with anterior thalamic involvement but sparing the midbrain). Percheron first described this variant in the 1970s, providing a foundational description of the arterial supply to the human thalamus (7).

In this context, the presence of a unilateral fPCA provides additional anatomical support for an AOP-related mechanism. In unilateral fPCA configurations, the contralateral P1 segment is often hypoplastic, thus suggesting that a single dominant arterial supply may perfuse both of the paramedian thalami. This arrangement may favor a unified mechanism, such as AOP occlusion. However, alternative mechanisms, including independent embolic occlusion of bilateral perforating arteries, cannot be completely excluded, particularly in cases featuring basilar artery occlusion (8–10). It should be noted that the three cases described in this report represent heterogeneous vascular pathologies, including basilar artery occlusion and PCA/vertebral artery stenosis. Therefore, our cases should not be interpreted as a single etiological entity. Rather, these cases illustrate how distinct upstream vascular abnormalities may converge to produce a similar pattern of bilateral paramedian thalamic infarction, suggestive of AOP involvement.

All of our patients presented with sudden coma and pupillary abnormalities; MRI/CT also confirmed bilateral thalamic infarction. According to the Lazzaro classification, Cases 1 and 2 were Type II (bilateral paramedian thalamic infarction without midbrain involvement), whereas Case 3 was Type I (bilateral paramedian thalamic infarction with midbrain involvement).

Vascular imaging demonstrated basilar artery occlusion in Cases 1 and 2, and severe left stenosis of the PCA in Case 3. A unilateral fPCA was identified in all cases. The bilateral patterns of thalamic infarction observed in this series were considered to be compatible with potential AOP involvement. However, given the presence of basilar artery occlusion in Cases 1 and 2, alternative mechanisms cannot be excluded, such as the bilateral embolic occlusion of PCA P1 perforators.

Embryologically, while the PCA typically matures as the terminal branch of the basilar artery supplying the occipital lobe, it originates from the internal carotid artery (ICA) via the posterior communicating artery (PComA) during early development. A persistent fPCA occurs in approximately 10% of individuals, in which the PCA remains predominantly supplied by the ICA via a prominent PComA; this condition is frequently associated with hypoplasia or aplasia of the ipsilateral P1 segment (11). Zhang et al. used high-resolution MRI to demonstrate that the configuration of the fPCA can alter hemodynamic processes and is prone to causing atherosclerosis and occlusion (12). This mechanism could be responsible for the pathology observed in Case 3.

The management of AOP infarction adheres to acute ischemic stroke protocols, including antiplatelet/antithrombotic therapy, statin therapy, blood pressure optimization, and neuroprotective measures. Endovascular therapy (EVT) may be considered in cases associated with large vessel occlusion (e.g., basilar or PCA). Evidence relating to the use of EVT for PCA occlusion remains heterogeneous; some studies suggest a clinical benefit (13), while others identify thalamic/midbrain involvement as a predictor of poor post-EVT outcomes (14). Recent case-based evidence has also demonstrated that timely thrombolysis or endovascular intervention may lead to favorable outcomes in selected patients with AOP infarction (15). The PLATO study reported that EVT was beneficial for cases with isolated PCA occlusion and an increased risk of hemorrhage (16). In our case series, Cases 1 and 2 underwent thrombectomy for occlusion of the basilar artery, whereas Case 3 received angioplasty for stenosis of the vertebral artery. The selection of EVT varied across cases based on underlying vascular pathology and anticipated treatment targets. In Cases 1 and 2, basilar artery occlusion or near-occlusion provided a clear indication for mechanical thrombectomy, with the primary goal of restoring forward flow in the posterior circulation and improving consciousness. Complete reperfusion was achieved in both cases (TICI grade 3), although clinical outcomes varied.

The situation in Case 3 was different. Although we identified severe stenosis of the left PCA P1 segment, we did not perform direct intervention on this segment. This decision was based on the relatively small vessel caliber and the higher procedural risk associated with manipulation in this region, including the potential for vessel injury and symptomatic hemorrhage. Instead, treatment was directed at improving posterior circulation inflow via angioplasty of the vertebral artery.

Differences in the clinical outcomes of our three cases may be explained by several factors. Case 3 involved midbrain infarction, which has been associated with poorer outcomes in posterior circulation stroke. Case 1 was treated for a longer period and was advanced in age, both of which may have contributed to the unfavorable prognosis. In contrast, Case 2 achieved timely reperfusion and was relatively younger; these factors may have contributed to a more favorable recovery.

Coma at onset is a consistently reported poor prognostic indicator in AOP infarction, with substantial proportions of patients failing to regain consciousness (17). Therapeutic options for persistent coma remain limited.

Several limitations of this study should be acknowledged. First, the small sample size (three cases) limits the generalizability of our findings. Second, the diagnosis of AOP infarction was inferred, as direct visualization of the artery is not feasible with current imaging techniques. Although characteristic imaging patterns and the presence of a unilateral fPCA provide a supportive anatomical context, alternative mechanisms cannot be definitively excluded. Third, the cases included in this study involve heterogeneous vascular pathologies and treatment strategies, including occlusion of the basilar artery and stenosis of the PCA/vertebral artery; this may have influenced both management decisions and clinical outcomes. These factors should be considered when interpreting the findings of this study.

## 4 Conclusion

Bilateral thalamic infarction secondary to AOP occlusion represents a rare and diagnostically challenging entity. Clinicians should be suspicious when encountering patients with acute impairment of consciousness, pupillary abnormalities, and vertical gaze palsy, particularly in the absence of significant limb weakness. While toxic, metabolic, and inflammatory etiologies warrant consideration, prompt neuroimaging, particularly MRI, is critical for timely diagnosis. The identification of a unilateral fPCA serves as a supportive, but non-specific, anatomical context for an AOP variant in cases of bilateral paramedian thalamic infarction (with or without midbrain involvement). Early recognition is paramount. Although endovascular intervention may be pursued with concurrent large vessel occlusion, overall prognosis remains guarded, especially in comatose patients.

## Data Availability

The original contributions presented in the study are included in the article/Supplementary Material; further inquiries can be directed to the corresponding author.
